# DNA Based Vaccine Expressing SARS-CoV-2 Spike-CD40L Fusion Protein Confers Protection Against Challenge in a Syrian Hamster Model

**DOI:** 10.3389/fimmu.2021.785349

**Published:** 2022-01-12

**Authors:** Levi A. Tamming, Diana Duque, Anh Tran, Wanyue Zhang, Annabelle Pfeifle, Emmanuel Laryea, Jianguo Wu, Sathya N. Thulasi Raman, Caroline Gravel, Marsha S. Russell, Anwar M. Hashem, Reem M. Alsulaiman, Rowa Y. Alhabbab, Jun Gao, David Safronetz, Jingxin Cao, Lisheng Wang, Wangxue Chen, Michael J. W. Johnston, Simon Sauve, Michael Rosu-Myles, Xuguang Li

**Affiliations:** ^1^ Centre for Biologics Evaluation, Biologic and Radiopharmaceutical Drugs Directorate, Health Products and Food Branch, Health Canada and World Health Organization Collaborating Center for Standardization and Evaluation of Biologicals, Ottawa, ON, Canada; ^2^ Department of Biochemistry, Microbiology and Immunology, Faculty of Medicine, University of Ottawa, Ottawa, ON, Canada; ^3^ Human Health Therapeutics Research Center, National Research Council of Canada, Ottawa, ON, Canada; ^4^ Vaccines and Immunotherapy Unit, King Fahd Medical Research Center, King Abdulaziz University, Jeddah, Saudi Arabia; ^5^ Department of Medical Microbiology and Parasitology, Faculty of Medicine, King Abdulaziz University, Jeddah, Saudi Arabia; ^6^ Department of Medical Laboratory Technology, Faculty of Applied Medical Sciences, King Abdulaziz University, Jeddah, Saudi Arabia; ^7^ National Microbiology Laboratory, Public Health Agency of Canada, Winnipeg, MB, Canada; ^8^ Department of Chemistry, Carleton University, Ottawa, ON, Canada

**Keywords:** SARS-CoV-2, coronavirus, vaccination, DNA, antibody response, pathology

## Abstract

SARS-CoV-2 infections present a tremendous threat to public health. Safe and efficacious vaccines are the most effective means in preventing the infections. A variety of vaccines have demonstrated excellent efficacy and safety around the globe. Yet, development of alternative forms of vaccines remains beneficial, particularly those with simpler production processes, less stringent storage conditions, and the capability of being used in heterologous prime/boost regimens which have shown improved efficacy against many diseases. Here we reported a novel DNA vaccine comprised of the SARS-CoV-2 spike protein fused with CD40 ligand (CD40L) serving as both a targeting ligand and molecular adjuvant. A single intramuscular injection in Syrian hamsters induced significant neutralizing antibodies 3-weeks after vaccination, with a boost substantially improving immune responses. Moreover, the vaccine also reduced weight loss and suppressed viral replication in the lungs and nasal turbinates of challenged animals. Finally, the incorporation of CD40L into the DNA vaccine was shown to reduce lung pathology more effectively than the DNA vaccine devoid of CD40L. These results collectively indicate that this DNA vaccine candidate could be further explored because of its efficacy and known safety profile.

## Introduction

Since its emergence in late 2019, the severe acute respiratory syndrome-coronavirus-2 (SARS-CoV-2) has caused one of the greatest pandemics in modern history, with over 215 million confirmed infections and 4.5 million deaths (1). This global health crisis has resulted in an unprecedented push to develop safe and efficacious vaccines against SARS-CoV-2. According to the World Health Organization (WHO), there are over 180 vaccines currently in pre-clinical development, with more than 100 having begun clinical testing (2). While more traditional vaccine technologies such as subunit and inactivated virus vaccines make up a large portion of this figure, many innovative vaccines strategies have been at the forefront of global vaccination campaigns, receiving emergency use authorization from multiple regulatory agencies. Lipid nanoparticle–formulated messenger RNA (mRNA) vaccines are one strategy that has seen widespread usage throughout the pandemic. Two mRNA vaccines in particular, Moderna’s mRNA-1273 (Spikevax) (3, 4) and Pfizer/BioNTech’s BNT162b2 (Comirnaty) (5, 6) have proven to be extremely safe and effective at preventing COVID-19 illness. With many leading regulatory bodies approving the Comirnaty vaccine (7–9), we are witnessing the beginning of a new era in vaccinology.

While unquestionably effective, the high cost and cold-storage requirements of mRNA vaccines impedes their use in both lower income countries and remote and isolated communities. Alongside protein subunit and inactivated virus vaccines, DNA vaccines present an invaluable alternative to mRNA vaccines due to their superior thermostability and reduced cost of production (10–12). Two prominent DNA vaccines against SARS-CoV-2 include Inovio pharmaceuticals’ INO-4800 and Zydus Cadila’s ZyCoV-D candidate vaccines, which both elicited strong humoral and cellular immune responses in their respective Phase I clinical trials (13, 14). In a first for a DNA-based SARS-CoV-2 vaccine, ZyCoV-D recently received emergency use approval in India (15).

Despite promising results, some concerns remain about DNA vaccine technologies, notably their low immunogenicity and subsequent ability to produce effective immune responses. One well-tested strategy to enhance the humoral and cell-mediated immune responses to DNA vaccines is *via* the inclusion of the cluster of differentiation 40 (CD40) ligand (CD40L) as a molecular adjuvant (16–22). CD40, a member of the TNF-receptor superfamily, is constitutively expressed in antigen-presenting cells (APCs) as a key regulator of their activation (23–25). The CD40-CD40L interaction represents one of the most critical steps in transitioning from the innate to the adaptive immune response. Our group has previously described the benefits of using CD40L as an adjuvant for vaccines against influenza (26), respiratory syncytial virus (27) and recently, Middle East respiratory virus (MERS-CoV) (28).

Given its previously demonstrated effectiveness in inducing strong and long-lasting immune response and specifically its ability to improve the safety of a vaccine against another coronavirus, we employed CD40L as an adjuvant to develop a DNA vaccine against SARS-CoV-2. To this end, we generated a pcDNA3.1-vectored vaccine encoding a secreted pre-fusion stabilized form of the SARS-CoV-2 spike protein fused to hamster CD40L *via* a trimerization motif. The immunogenicity and protective efficacy of this vaccine candidate was evaluated in a Syrian hamster challenge model.

## Materials and Methods

### Cell Lines and Viruses

BHK-21, HEK293T and HEK293T-ACE2 cells were cultured in Dulbecco’s Modified Eagle Medium (DMEM) supplemented with 25 mM HEPES, 20 U/mL Penicillin, 0.02 mg/mL Streptomycin and 10% heat-inactivated fetal-bovine serum (FBS). HEK-Blue™ CD40L cells were cultured in DMEM supplemented with 20 U/mL Penicillin, 0.02 mg/mL Streptomycin, 100 µg/mL Normocin and 10% heat-inactivated FBS. Vero cells were cultured in DMEM supplemented with 1X non-essential amino acid, 20 U/mL Penicillin, 0.02 mg/mL Streptomycin, 1 mM sodium pyruvate and 10% heat-inactivated FBS. SARS-CoV-2 isolate Canada/ON/VIDO-01/2020 was propagated on Vero E6 cells and titered on Vero cells. Exact genetic identity to original isolate was confirmed by whole viral genome sequencing. Passage three virus stocks were used in all subsequent experiment that required live virus.

### DNA Vaccines

DNA sequences encoding the SARS-CoV-2 isolate Wuhan-Hu-1 spike (GenBank accession #MN908947) ectodomain (residues 1–1208) fused to a T4 fibritin foldon trimerization motif (YIPEAPRDGQAYVRKDGEWVLLSTFLG) without (S.dTM.PP) or with the ectodomain of *Mesocricetus auratus* CD40L (S.dTM.PP-CD40L) (GenBank accession #XM_005084522.4, residues 118-260) were commercially synthesized (BioBasic, Toronto, ON). Domains were separated by flexible glycine-serine linkers sequences “GSGG”. The S ectodomains were prefusion stabilized *via* a “GSAS” substitution at the furin cleavage site (residues 682-685) and proline substitutions at residues 986 and 987 as previous reported (29). Coding sequences were codon optimized for expression in Syrian hamsters and subcloned into the mammalian expression plasmid pcDNA3.1 (+) using *KpnI* and *NotI* restriction enzymes (**Supplemental Figure 1**). Bulk DNA vaccine preparations were prepared with endotoxin-free gigaprep kits (Qiagen, Hilden, Germany) and the sequences were validated with Sanger sequencing.

### *In Vitro* Protein Expression

HEK293T cells were transiently transfected in 6-well plates with 1.6 µg of pcDNA3.1, pcDNA3.1 S.dTM.PP or pcDNA3.1 S.dTM.PP-CD40L using Lipofectamine™ 3000 Transfection Reagent (ThermoFisher, Ottawa, ON) according to the manufacturer’s instructions and incubated for 48 hours at 37°C, 5% CO_2_. The cells were washed with phosphate-buffered saline (PBS) and then lysed with radioimmunoprecipitation assay buffer (ThermoFisher, Ottawa, ON). Lysates were electrophoresed on a 4-15% TGX stain-free SDS-PAGE gel (Bio-Rad, Saint-Laurent, QC) and subsequently transferred to a polyvinylidene difluoride membrane. Membranes were blocked for 1h at room temperature with tris-buffered saline (TBS) containing 0.5% Tween 20 (Sigma-Aldrich, St. Louis, MO) (V/V) (TBS-T) and 5% (W/V) non-fat milk powder then incubated overnight at 4°C in blocking buffer containing either polyclonal rabbit anti-SARS-CoV-2 Spike antibody (1:3000 dilution) (Sino Biological) or polyclonal rabbit anti-β-actin antibody (1:1000 dilution) (Cell Signaling). Membranes were then incubated for 1 hour at room temperature with goat anti-rabbit horseradish peroxidase (HRP)-conjugated secondary antibody (1:75, 000 dilution) (ThermoFisher, Ottawa, ON) in blocking buffer and developed using SuperSignal™ West Femto Maximum Sensitivity Substrate (ThermoFisher, Ottawa, ON) and a ChemiDoc MP imaging system (Bio-Rad, Saint-Laurent, QC).

### CD40L Bioactivity Assay

HEK293T cells were either mock transfected or transiently transfected in a 24-well plate with 1 µg of pcDNA3.1, pcDNA3.1-S.dTM.PP or pcDNA3.1-S.dTM.PP-CD40L using Lipofectamine™ 3000 Transfection Reagent (ThermoFisher, Ottawa, ON) and incubated for 24 hours at 37°C, 5% CO_2_. In a 96-well plate, 100 µL of growth media from the transfected cells was mixed with 100 µL of HEK-Blue CD40L cells (InvivoGen, San Diego, CA) resuspended at 2.0×10^5^ cells per mL in fresh media. Following a 24-hour incubation at 37°C in a 5% CO_2_ incubator, 20 µL of cell culture media was mixed with 180 µL of QUANTI-Blue™ Reagent in a 96-well plate. The absorbance at 630 nm was measured periodically after a 30-minute incubation at 37°C using a Synergy™ 2 microplate reader (BioTek, Winooski, VT).

### Hamster Immunization

6-8 week old female Syrian hamsters were purchased from Charles River Laboratories (Saint-Constant, Canada). Animal experiments were approved by the National Research Council Canada (NRC) Human Health Therapeutics Animal Care Committee. Animal procedures were performed by trained staff in accordance with regulations and guidelines by the Canadian Council on Animal Care and the NRC Human Health Therapeutics Animal Care Committee. All infectious work was carried out under ABSL-3 conditions at the NRC. Animals were randomly allocated into three different experimental groups (n=12 per group) and were immunized twice with 100 µg of pcDNA3.1, pcDNA3.1 S.dTM.PP or pcDN3.1 S.dTM.PP-CD40L on days 0 and 28. The DNA vaccines were suspended in PBS at a concentration of 1 mg/mL and administered intramuscularly in the hamster’s left tibialis anterior muscle with a needle syringe. Hamster serum was collected on days -7, 21 and 42. On day 49 the hamsters were intranasally challenged with 1.0×10^5^ PFU of SARS-Co-2 (Canada/ON/VIDO-01/2020). Animals were euthanized by CO_2_ either 2- or 7-days post-challenge and the nasal turbinate, lung and spleen were collected for determination of viral titers and histopathology analysis.

### ELISA

Nunc MaxiSorp™ flat-bottom 96-well plates (ThermoFisher, Ottawa, ON) were coated with 1 µg/mL of either SARS-CoV-2 Spike S1+S2 ECD-His recombinant protein or SARS-CoV-2 Spike RBD-His recombinant protein (Sino Biological, Beijing, China) in PBS and incubated overnight at 4°C. Plates were washed with PBS containing 0.1% Tween-20 (PBS-T) before blocking with 3% (w/v) Bovine Serum Albumin (IgG-Free, Protease-Free) (Jackson Immuno Research, West Grove, PA) in PBS-T for 2 hours at 37°C. The plates were washed again and two-fold serial dilutions of hamster serum, starting from 1:50 up to 1:102400 were added to the wells and incubated for 1 hour at 37°C. Plates were then washed with PBS-T and Peroxidase AffiniPure Goat Anti-Syrian Hamster IgG (H+L) (Jackson Immuno Research, West Grove, PA) was added to each well at 1:4000 and incubated at 37°C for 1h. Plates were washed again with PBS-T and 100 µL of Tetramethylbenzidine (TMB) substrate (Cell Signaling Technology, Danvers, MA) was added to each well. After a two-minute incubation at room temperature, 100 µL of 0.16 M sulfuric acid was added to terminate the reaction and absorbance was measured at 450 nm. Endpoint titers were expressed as the reciprocals of the final detectable dilution with an OD above the cut-off value, which was defined as the average OD of the pcDNA3.1-empty samples plus 3 standard deviations.

### Pseudovirus Neutralization Assay

The neutralizing activity of vaccinated hamster sera was determined using a luciferase reporter SARS-CoV-2 S pseudovirus described previously (30). Briefly, pseudotyped VSV was generated by concurrently infecting HEK293T cells with G*ΔG-VSV (Kerafast, Winston-Salem, NC) and transfecting them with pCDNA3.1 encoding either SARS-CoV-2 S from the Wuhan-1 or B.1.351 (31) lineages or SΔCT from the B.1.617.2 lineage. Cell culture supernatant containing the pseudovirus was collected 24- and 48-hours post-infection before being mixed and purified by filtration through a 0.45 µm filter. In a 96-well plate, serum samples heat-inactivated at 56°C for 30 mins were serially diluted three-fold, mixed with 50 µL of pseudovirus diluted to 1.3×10^4^ TCID_50_/mL and incubated for 1h at 37°C, 5% CO_2_. Afterwards 100 µL of 2×10^5^ cells/mL of HEK293T-ACE2 was added to each well. Following an additional 24h incubation, 150 µl of supernatant was aspirated and replaced with 100 µL of Bright-Glo luciferase reagent (Promega, Madison, WS). Luminescence was measured using a Synergy™ 2 microplate reader (BioTek, Winooski, VT). The 50% neutralization titers (NT50) were determined as previously reported (30), where the NT50 was the reciprocal of the sample dilution at which a 50% reduction in relative light units (RLU) was observed relative to the average of the no-serum control wells.

### Lung Viral Titration Assay

Plaque assays were performed under biosafety level-3 (BSL-3) conditions. Left lung tissues were weighed and then homogenized in 1 mL of PBS. The homogenates were centrifuged and the clarified supernatants were used in a plaque assay. In brief, a 1:10 serial dilution of clarified lung homogenate was made in infection media (DMEM supplemented with 1X non-essential amino acid, 20 U/mL Penicillin, 0.02 mg/mL Streptomycin, 1 mM sodium pyruvate, and 0.1% bovine serum albumin). Virus was adsorbed on Vero cells at 37°C and 5% CO_2_ for 1h before the inoculum was removed and overlay media was added (1X infection media with 0.6% ultrapure, low-melting point agarose). The infection was incubated at 37°C and 5% CO_2_ for 72h, then fixed with 10% formaldehyde and stained with crystal violet. Plaques were enumerated and PFU was determined per gram of lung tissue.

### Subgenomic mRNA Assay

SARS-CoV-2 E subgenomic mRNA (sgmRNA) levels in lungs and nasal turbinates were assessed by RT-qPCR using previously described TaqMan probes (32). SARS-CoV-2 E sgmRNA for use as a standard curve was transcribed from a commercially synthesized pcDNA3.1 E sgmRNA vector (BioBasic, Toronto, ON) using a TranscriptAid T7 High Yield Transcription Kit (ThermoFisher, Ottawa, ON) according to the manufacturer’s protocol. Lung tissues were placed into RNA shield buffer (Zymo Research, Irvine, CA) and incubated overnight at 4°C to allow for reagent penetration before freezing at -80°C. Viral RNA was extracted under BSL-3 conditions from the mechanically homogenized samples using a Quick-RNA Viral Kit (Zymo Research, Irvine, CA). Inactivated purified viral RNA was then removed from the ABSL-3 facility for subsequent qRT-PCR experiments. sgmRNA levels were assessed using a TaqMan custom gene expression assay (ThermoFisher, Ottawa, ON) (**Table 1**) and a one-step Fast Virus master mix (ThermoFisher, Ottawa, ON) according to the manufacturer’s protocol. RT-qPCR reactions were conducted using an Applied Biosystems™ 7500 Fast Real-time PCR instrument. Standard curves of *in vitro* transcribed sgmRNA were used to calculated sgmRNA copiers per mL.

**Table 1:** E sgmRNA primers.

Name	Sequence
Leader_F	5’- CGATCTCTTGTAGATCTGTTCTC-3’
E_Probe	5’- ACACTAGCCATCCTTACTGCGCTTCG-3’
E_Rev	5’-FAM-ATATTGCAGCAGTACGCACACA-MGB- 3’

### Histopathology

Right lungs were collected for histopathology analysis. The tissues were fixed for 72h in 10% neutral buffered formalin and processed by standard paraffin embedding methods (33). Sections were cut 4 µm thick, stained with hematoxylin-eosin (HE), and examined under microscopy. The severity and extent of pneumonia (the presence of inflammatory polymorphonuclear and mononuclear cells) was scored blinded by a veterinarian pathologist based on the criteria of Lien et al. (34) with modifications (**Table 2**).

**Table 2:** Histological Scoring Criteria.

Score	Histological changes
0	No significant finding
1	Minor peribronchial/bronchiolar and perivascular inflammation with slight thickening of alveolar septa with small numbers of mononuclear cell infiltration
2	Apparent inflammation and alveolus septa thickening with more interstitial mononuclear inflammatory infiltration; focal areas of consolidation
3	Multiple focal consolidation with alveolar septa thickening, and increased infiltration of inflammatory cells
4	Area of consolidation with extensive alveolar septa thickening, collapse of alveoli, restricted fusion of the thick septa, and more cell infiltration in alveolar space and the areas surrounding airways and blood vessels
5	As 4, but the lung is almost completely consolidation

### Statistical Analysis

Normality of the study data was assessed by a Shapiro-Wilk test (alpha-level=0.05). Whenever data or their log transformations were deemed not of normal distribution, a non-parametric approach was adopted. A Kruskal-Wallis H test with Holm’s sequential Bonferroni adjustment was applied for pairwise (between-group) comparisons of S- and RBD-specific IgG endpoint titers, neutralizing antibody titers, lung viral burden and histology scores. A one-way analysis of variance (ANOVA) with Bonferroni’s adjustment was applied for pairwise (between-group) comparisons of CD40L bioactivity, weight loss data by day, nasal viral titer and lung subgenomic mRNA. The abovementioned analyses were performed using either SAS Enterprise Guide 7.1 or GraphPad PRISM 7. * p < 0.05, **p < 0.01, ***p < 0.001, ****p <0.0001.

## Results

### Recombinant Antigen Design, Expression, and Bioactivity

**Figure 1:**
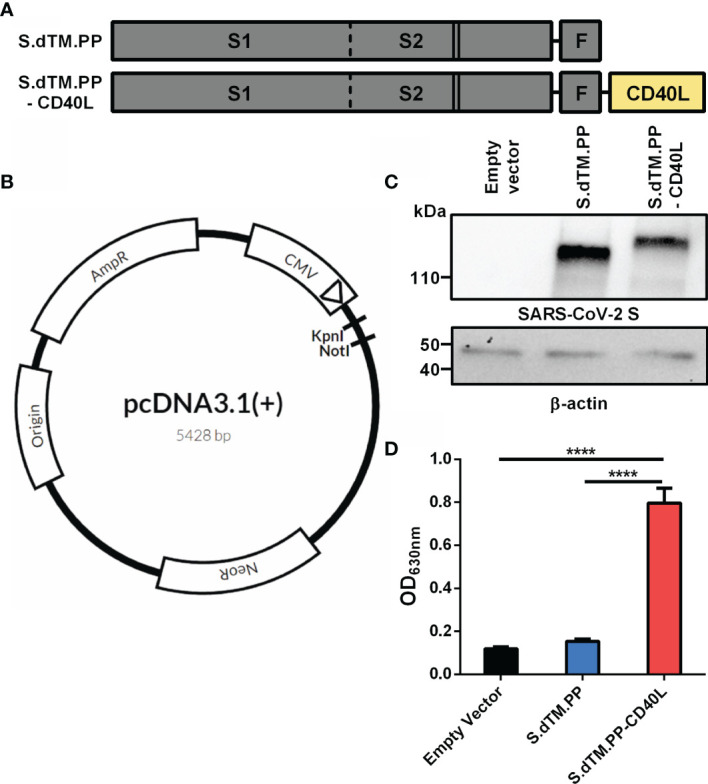
Spike-CD40L fusion antigen design and vaccine characterization. **(A)** The DNA vaccine antigens were based on a truncated SARS-CoV-2 spike protein lacking the transmembrane domain and C-terminal tail. The S protein was prefusion stabilized *via* the introduction of two stabilizing proline mutations (solid lines) and the replacement of the furin cleavage site (dotted line). The S protein was fused to a T4 fibritin trimerization motif (F) with or without the ectodomain of CD40L. **(B)** Antigens were subcloned into pcDNA3.1 (+) vector using KpnI and NotI restriction sites. **(C)** Antigen expression was detected in BHK-21 cells transfected with the DNA vaccines. Empty pcDNA3.1 was used as a negative control and β-actin expression was used as a loading control. kDa, kilodalton. **(D)** CD40L reporter HEK293 cells were stimulated for 24h with media collected from HEK293T cells transfected with the DNA vaccines. SEAP expression in the cell culture supernatant post-24h incubation was measured using QUANTI-Blue™ reagent. Abs_630nm_ values were measured after a 30-minute incubation. Data shown is mean ± SEM; n = 3 per group. ****p < 0.0001.

Recombinant C-terminally truncated pre-fusion stabilized SARS-CoV-2 S proteins (S.dTM.PP) without or with a fused CD40L ectodomain (S.dTM.PP-CD40L) were generated in pcDNA3.1 vectors (**Figures 1A, B**). Western blot analysis was used to confirm the *in vitro* expression of both S.dTM.PP and S.dTM.PP-CD40L in transfected BHK-21 cells (**Figure 1C**). The western blot revealed single bands for both the S.dTM.PP and S.dTM.PP-CD40L constructs near their expected molecular weights (MW) of 137 and 152 kDa respectively. Next, a cell-based CD40 secreted embryonic alkaline phosphatase (SEAP) reporter assay was used to ensure that the fused CD40L ectodomain remained biologically active and capable of engaging with CD40 (**Figure 1D**). Cell culture media from HEK293T cells transfected with the DNA vaccines was transferred onto reporter HEK-Blue CD40L cells to test the engagement of vaccine antigen derived CD40L with CD40 from HEK-Blue cells. The S.dTM.PP-CD40L construct induced significantly higher levels CD40-CD40L signaling than the other two constructs (**Figure 1C**), confirming the bioactivity of the fused CD40L ectodomain.

### DNA Vaccines Elicit Strong Humoral Responses in Syrian Hamsters

**Figure 2:**
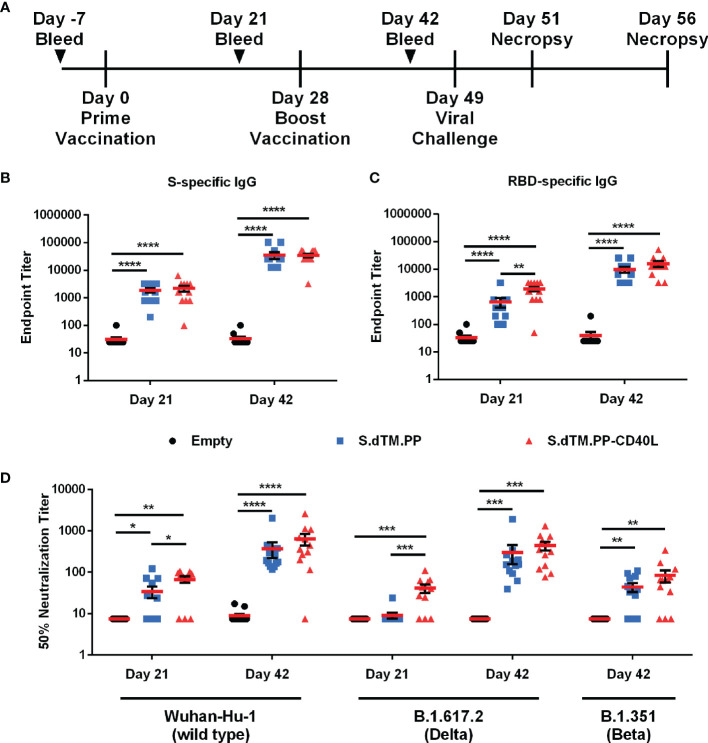
DNA vaccines induce robust humoral response. **(A)** Female Syrian hamsters were randomly divided into three experimental groups (n= 12) and immunized intramuscularly on day 0 and 28 with 100 µg of pcDNA3.1, pcDNA3.1 S.dTM.PP or pcDNA3.1 S.dTM.PP-CD40L. Animals were challenged intranasally with 1×10^5^ PFU of SARS-CoV-2 on day 49 and euthanized 2- and 7-days post-infection (dpi). Immunoglobulin determination of total spike **(B)** and RBD **(C)** -specific IgG in the sera of immunized hamsters was done on days 21 and 42. **(D)** The 50% neutralization titer of immunized hamster sera at day 21 and day 42 was determined using wild type, B.1.671.2 and B.1.351 SARS-CoV-2 spike pseudotyped-VSV. Data shown is mean ± SEM; n = 12 per group. *p < 0.05, **p < 0.01, ***p < 0.001, ****p < 0.0001.

Female Syrian hamsters were immunized with two 100 μg doses of pcDNA3.1-S.dTM.PP, pcDNA3.1-S.dTM.PP-CD40L or empty pcDNA3.1. The vaccines were administered intramuscularly in PBS at days 0 and 28 (**Figure 2A**). Binding antibodies against the full-length SARS-CoV-2 S (**Figure 2B**) and RBD (**Figure 2C**) were quantified 21 and 42 days after prime vaccination using an indirect ELISA. At day 21 following a single administration, S.dTM.PP-CD40L induced significantly higher antibody titers against SARS-CoV-2 RBD than its non-fusion counterpart, S.dTM.PP (**Figure 2C**). Following the boost vaccination, both vaccines elicited similar antibody titers against both full-length S and RBD (**Figures 2B, C**), at higher levels than what was observed for both on day 21. The neutralizing antibody (NAb) titer of serum collected on either day 21 or day 42 was determined using a VSV-based pseudovirus neutralization assay (**Figure 2D**). Coinciding with the increased RBD-specific IgG, after a single dose, the S.dTM.PP-CD40L vaccine induced a greater 50% neutralization titer against wild type (WT) and B.1617.2 variant pseudotyped-VSV than the S.dTM.PP vaccine (**Figure 2D**). After the boost vaccination, both spike vaccines induced significant NAb responses against WT, B.1.351 and B.1617.2 pseudotyped VSV (**Figure 2D**).

### DNA Vaccines Protect Hamsters From Weight Loss and Reduce Viral Burden

**Figure 3:**
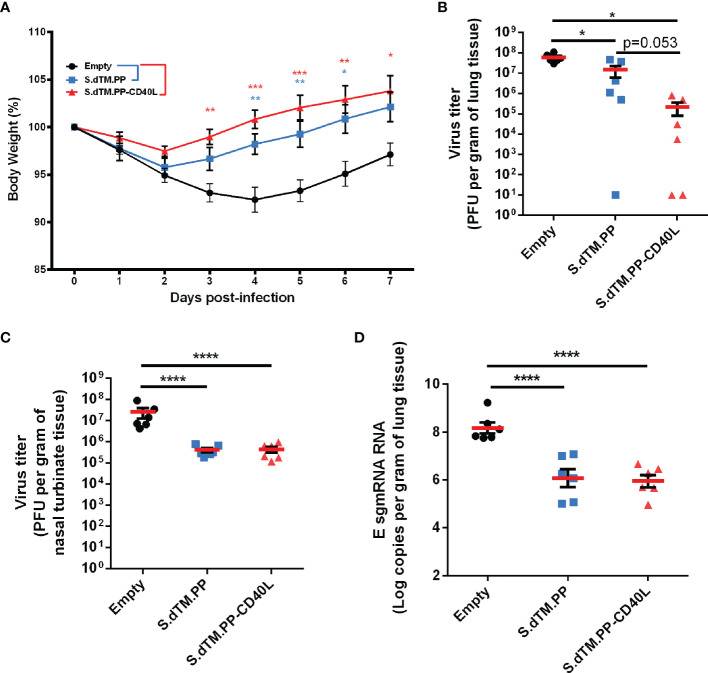
DNA vaccines reduce viral loads and improve weight recovery. **(A)** Syrian hamster body weight was measured for 7-days following viral challenge (n=6). Viral load in the lungs **(B)** and nasal turbinates **(C)** of SARS-CoV-2 challenged hamsters on day 2 post-infection (n=6). **(D)** Number of E sgmRNA copies in the lungs was determined *via* RT-qPCR 2 days post-infection (n=6). Data shown is mean ± SEM; n = 6 per group. *p < 0.05, **p < 0.01, ***p < 0.001, ****p < 0.0001.

On day 49, animals were challenged intranasally with 1×10^5^ PFU of SARS-CoV-2 (**Figure 2A**). Changes in body weight were monitored daily post-challenge (**Figure 3A**) until animals were euthanized either 2 or 7 days post-challenge. On average, animals in the empty vector control group continued to lose body weight for 4 days post-challenge, reaching a maximum weight loss of 7.6% on day 4. Comparatively, animals immunized with the S.dTM.PP-CD40L and S.dTM.PP vaccines began to recover weight much earlier post-challenge, beginning to have significantly higher body weights than the control animals on days 3 and 4 respectively (**Figure 3A**). Viral burden in the respiratory tissues of Syrian hamsters was assessed by both plaque assay and RT-qPCR quantification of SARS-CoV-2 subgenomic mRNA (sgmRNA). On day 2 post-infection, the S.dTM.PP and S.dTM.PP-CD40L groups had significantly reduced viral burden in both lung and nasal turbinates compared to the empty vector control (**Figures 3B, C**). Although not statistically significant, there was a notable trending difference (p=0.053) of more than 50 folds in the mean lung viral titers between S.dTM.PP (1.4×10^7^ PFU/g) and S.dTM.PP-CD40L (2.2×10^5^ PFU/g). Vaccination with both S.dTM.PP and S.dTM.PP-CD40L significantly reduced the number of E sgmRNA copies in the hamster lungs 2 dpi (**Figure 3D**) relative to the empty vector control. Plaque forming units and E sgmRNA levels were below the limit of detection for all groups 7 days post-challenge (data not shown).

### DNA Vaccine Expressing S-CD40L Fusion Protein Most Effectively Reduced Lung Pathology Following SARS-CoV-2 Challenge

**Figure 4:**
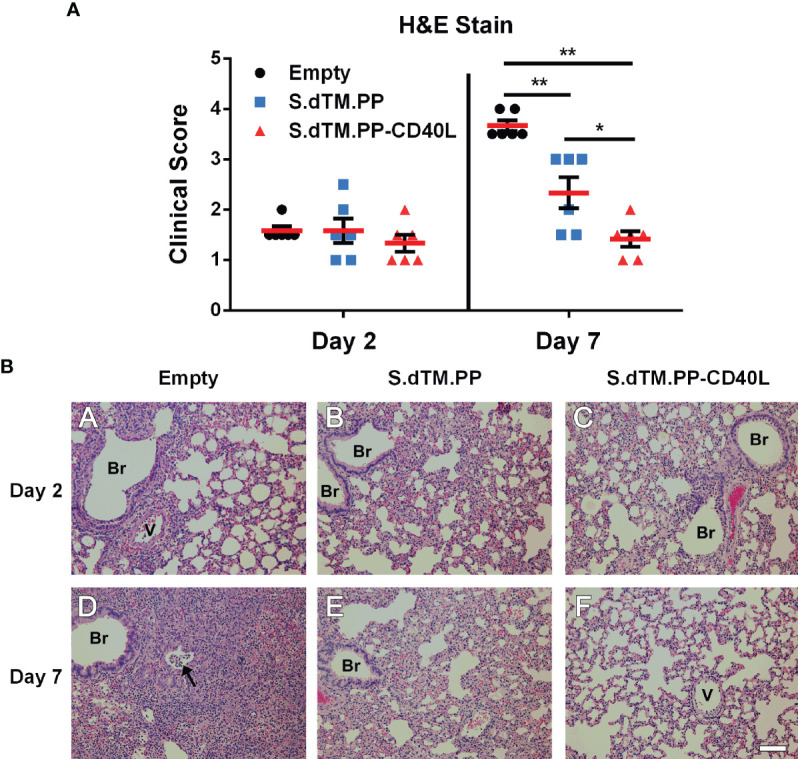
Lung Pathology following SARS-CoV-2 Challenge. **(A)** Summary of histopathological scores. Data shown is mean ± SEM; n = 6 per group. *p < 0.05, **p < 0.01. **(B)** Representative photomicrographs of lung histopathology in SARS-CoV-2-infected hamsters. Groups of female golden Syrian hamsters (n=6) were intramuscularly immunized with pcDNA3.1 S.dTM.PP, pcDNA3.1 S.dTM.PP-CD40L or empty vector as a control on day 0 and 28. The hamsters were intranasally challenged with 1.0×10^5^ PFU of SARS-CoV-2 on day 49 and sacrificed 2 or 7 days later. (A-C) Lung histopathology from infected hamsters killed at day 2 post-challenge. The lungs from hamsters vaccinated with empty vector (A), S.dTM.PP (B), and S.dTM.PP-CD40L (C) showed mild to moderate interstitial pneumonia of similar severity. (D-F) Lung histopathology from infected hamsters killed at day 7 post-challenge. (D) The lung from a hamster vaccinated with the empty vector showed areas of consolidation with the occasional presence of mixed inflammatory cells in the bronchiolar lumen (arrow). (E) The lung from a hamster vaccinated with S.dTM.PP showed apparent mononuclear inflammatory infiltration in the alveolar septa and focal areas of consolidation. (F) The lung from a hamster vaccinated with S.dTM.PP-CD40L showed only mild interstitial pneumonia that is milder than that in the hamster vaccinated with S.dTM.PP (E). Br, bronchioles; V, blood vessel. H&E. Bar = 100 µm.

Right lung lobes were collected both 2- and 7-days post-challenge for histopathological analysis. Lungs from all infected hamsters at day 2, regardless of administered vaccine, showed mild to moderate interstitial pneumonia consisting of small to moderate numbers of mononuclear cell infiltration, thickening of the alveolar septa, and occasional presence of mixed neutrophils and mononuclear cells in the airway lumen. In addition, we detected mild to moderate infiltration of mononuclear cells in some perivascular and peribronchial areas of the lung (**Figures 4A, B**). Substantial differences in the severity of lung histopathology were observed in the infected hamsters at day 7, depending on the type of vaccine received. As anticipated, hamsters vaccinated with the empty vector showed the most severe lung histopathology. They displayed areas of consolidation due to the extensive alveolar septa thickening, collapse of alveoli, and inflammatory cell infiltration in alveolar septa and the areas surrounding airways and blood vessels. Hamsters vaccinated with S.dTM.PP showed milder lung histopathology, which were comparable to those seen at day 2 but with more apparent mononuclear inflammatory infiltration in the alveolar septa and focal areas of consolidation. The hamsters vaccinated with S.dTM.PP-CD40L showed even milder lung histopathology in their lungs than the hamsters vaccinated with S.dTM.PP (**Figures 4A, B**), although the nature of the histopathological changes were similar between the two groups of hamsters. There were no overt abnormal changes in the nasal turbinate or spleen of any infected hamsters.

## Discussion

Many vaccine candidates against SARS-CoV-2 have been developed in an attempt to bring a halt to the COVID-19 pandemic. While vaccination efforts are underway across the globe, there remains a need for affordable and equitable vaccines. This need is heightened by the continued emergence of SARS-CoV-2 variants with increased resistance to neutralizing antibodies (35–37). These variants of concerns and potential waning immunological memory (38) may require administration of annual booster shots, exacerbating costs. DNA vaccines present a cost-effective and temperature-stable alternative to mRNA vaccines with similar immunological characteristics. Multiple DNA vaccines against SARS-CoV-2 have been tested in various animal models and clinical trials (13, 39–43). Intramuscular vaccination with 5 mg of naked pcDNA3.1 vectored vaccines encoding different variations of the SARS-CoV-2 spike protein, including S.dTM.PP, were shown to induce neutralizing antibodies and reduce levels of viral sgmRNA in the lungs of rhesus macaques (39). The pVAX-1-vectored ZyCoV-D vaccine showed strong humoral responses in mice, guinea pigs and rabbits when administered intradermally at 25 µg, 100 µg and 500 µg doses respectively (44). The ZyCoV-D vaccine was also found to be safe and immunogenic in a non-randomized phase I trial (14). INO-4800, a pGX0001-vectored Spike with an N-terminal IgE leader sequence displayed strong humoral and cell-mediated immune responses in mice and guinea pigs when administered intradermally using electroporation (43). The INO-4800 DNA vaccine was well-tolerated and immunogenic in all participants of a phase I clinical trial (13) and is now being tested in Phase II/III trials. INO-4800 is one of only a handful of candidate DNA vaccines currently undergoing clinical testing (**Supplemental Table 1**). As DNA vaccines appear poised to become a valuable tool against COVID-19, research into overcoming their limitations and improving the technology is more essential than ever.

In this work, we evaluated the protective efficacy of a pcDNA3.1 vectored SARS-CoV-2 spike antigen fused with and without CD40L in a SARS-CoV-2 Syrian hamster challenge model. It is noted in the literature that doses of DNA vaccines vary, ranging from 50 to 200 µg with or without the use of alternative DNA vaccine delivery methods (45–47). In this study, we employed a 100 µg dose, as our study was mainly intended to compare the vaccines with or without CD40 ligand. The pcDNA3.1 S.dTM.PP-CD40L vaccine was able to induce a significant antibody response after a single dose (**Figures 2B–D**). Notably after a single dose, the S.dTM.PP-CD40L vaccine generated higher RBD-specific IgG antibody titers than the spike vaccine devoid of CD40L and induced a significantly greater NAb response against WT and B.1617.2 pseudoviruses (**Figure 2D**). Two weeks following the second immunization, the two spike vaccines induced similarly robust humoral responses, including the significant induction of neutralizing antibodies against WT, B.1.351 and B.1.671.2 spike pseudotyped VSV (**Figure 2D**). Post-challenge all animals experienced some level of SARS-CoV-2 related pathology. Vaccination with either spike vaccine led to a quicker recovery of body weight (**Figure 3A**) and reduced lung and nasal viral burdens (**Figures 3B–D**) post-challenge relative to recipients of the empty vector. While the CD40L-adjuvanted DNA vaccine did not induce statistically significant differences when compared directly to its non-adjuvanted counterpart, earlier body weight recovery and a trending decrease in pulmonary viral burden were observed for S.dTM.PP-CD40L vaccinated animals. At 2 days post-challenge all animals had comparable lung histopathology (**Figure 4A**) despite differences in lung and nasal viral burden. This result is not unexpected, as the absence of strong mucosal immunity is likely to delay clearance of viral infection and resolution of pathological changes (48, 49). In this light, CD40L seemed to contribute significantly to the recovery from damage to the lower respiratory tract. Substantial differences were noted in lung histopathology at day 7 post-challenge, where hamsters vaccinated with S.dTM.PP-CD40L had milder pathology than both the empty vector and S.dTM.PP immunized animals (**Figure 4A**). The severe lung pathology at day 7 post-challenge in hamsters vaccinated with the empty vector (**Figure 4A**) but with no detectable viruses supports the notion that histopathology caused by the infection can persist for days after clearance of the SARS-CoV-2 infection (50).

While humoral responses and neutralizing antibodies play a critical role in vaccine-induced immunity against SARS-CoV-2 (51–53), it is important to consider strategies that also drive robust and long-lasting T cells responses. Despite not generating neutralizing antibodies, T cell epitope vaccines provide partial protection form SARS-CoV-2 challenge, suggesting T cell responses may also contribute to protection (54). Limited reagent availability for the Syrian hamster model precludes the comprehensive characterization of CD40L’s effect on immune subtypes and T cell responses without the use of an additional animal model. However, mechanistic explanations for the observed reduction in lung pathology can potentially be inferred from previous work. In the past, our group and others have demonstrated that the addition of CD40L enhances antigen-specific T cell responses and improves vaccine efficacy against various viruses (21, 26–28, 55–57). Notably in one study, immunization with an influenza nucleoprotein CD40L fusion vaccine provided no protection against RSV challenge in BALB/c mice (27). This result highlights the inability of CD40L alone to induce protective immune responses, with its beneficial effects rather being mediated through the enhancement of antigen-specific responses. In our previous study of recombinant adenovirus-5 vectored vaccines against MERS-CoV, despite affording similar reductions in viral burden as S1 alone, only S1-CD40L was able to prevent pulmonary perivascular hemorrhage post–MERS-CoV challenge in the transgenic Human Dipeptidyl Peptidase 4 Mouse Model (28). While pulmonary pathology did not manifest as perivascular hemorrhage in this study, owing to a variety of factors including the vaccine form, challenge virus and animal model, the reduced pulmonary pathology reported here aligns with these previous MERS-CoV findings, suggesting that a balanced protective immunity mediated by the CD40L fusion domain may have afforded additional protection from SARS-CoV-2 challenge.

Despite promising results, DNA vaccine adoption and utilization lags behind that of mRNA vaccines. Historically, the theoretical potential for DNA vaccines to integrate into the host genome has been of great concern; however, experimental evidence has shown the rate of integration to be below rates of spontaneous mutations (58, 59). Similar concerns also existed about the induction of anti-DNA antibodies, although numerous pre-clinical and clinical studies have practically dismissed this concern (60). One other major concern about DNA vaccines has been their historically poor therapeutic efficacy, driven partly by low immunogenicity and the inability of unformulated DNA vaccines to avoid DNase degradation and reach the nucleus. One potential avenue for improvement is through the use of alternative immunization devices, such as jet injectors, electroporation and gene-guns, all of which have been shown to improve the uptake of DNA vaccines and their subsequent efficacy relative to needle injection (61, 62). Another promising strategy is encapsulating the DNA vaccines in nanoparticles, which can improve DNA uptake, protect DNA from DNase degradation and act as an vaccine adjuvant (63–66). The successful usage of lipid-nanoparticle formulated RNA vaccines against SARS-CoV-2 lends credence to their use as a DNA vaccine delivery vector (67).

Our results underlie the need to further explore the safety and efficacy of DNA vaccines. We demonstrate the beneficial effect of using CD40L as a molecular adjuvant for a SARS-CoV-2 spike vaccine, significantly reducing lung pathology compared to a non-adjuvanted counterpart. Our work has its limitations. Specifically, while Syrian hamsters are one of the best small-animal models for vaccine evaluation against SARS-CoV-2, the scarcity of research reagents for the model precludes its use for mechanistic investigation. Further studies in mice may aim to characterize the effects of CD40L on T cell responses to determine potential molecular mechanisms underlying changes in disease pathology. Additional experiments may also investigate the potential synergism of this vaccine candidate with improved methods of DNA vaccine delivery such as lipid-nanoparticles. These experiments are currently ongoing in our laboratories.

## Data Availability Statement

The original contributions presented in the study are included in the article/**Supplementary Material**. Further inquiries can be directed to the corresponding authors.

## Ethics Statement

Animal experiments were reviewed and approved by the National Research Council Canada (NRC) Human Health Therapeutics Animal Care Committee.

## Author Contributions

LT, MR, DS, JC, LW, WC, MJ, SS, MR-M, and XL contributed to the conceptualization. LT, DD, AT, WC, WZ, AP, EL, JW, CG, MR, RMA, RYA, and WC conducted the experiments. LT, AT, AH, WC, WZ, AP, EL, JW, SR, RMA, RYA, and WC were involved in data curation and formal analysis. LT and JG performed the statistical analysis. AT, AH, DS, JC, LW, WC, MJ, SS, MR-M, and XL were involved in funding acquisition, supervision and project administration. LT wrote the first draft of the manuscript. WC and XL wrote sections of the manuscript. All authors contributed to manuscript revision, read and approved the submitted version.

## Funding

This work was funded by the Government of Canada.

## Conflict of Interest

The authors declare that the research was conducted in the absence of any commercial or financial relationships that could be construed as a potential conflict of interest.

## Publisher’s Note

All claims expressed in this article are solely those of the authors and do not necessarily represent those of their affiliated organizations, or those of the publisher, the editors and the reviewers. Any product that may be evaluated in this article, or claim that may be made by its manufacturer, is not guaranteed or endorsed by the publisher.
