# Supplementary material for: DNA Based Vaccine Expressing SARS-CoV-2 Spike-CD40L Fusion Protein Confers Protection Against Challenge in a Syrian Hamster Model
Source: Front Immunol. 2022 Jan 12;12:785349. doi: 10.3389/fimmu.2021.785349 (PMC8789660; doi:10.3389/fimmu.2021.785349)
Supplement: Supplementary file 1 [file DataSheet_1.pdf]

Supplementary Material

1 Supplementary Data

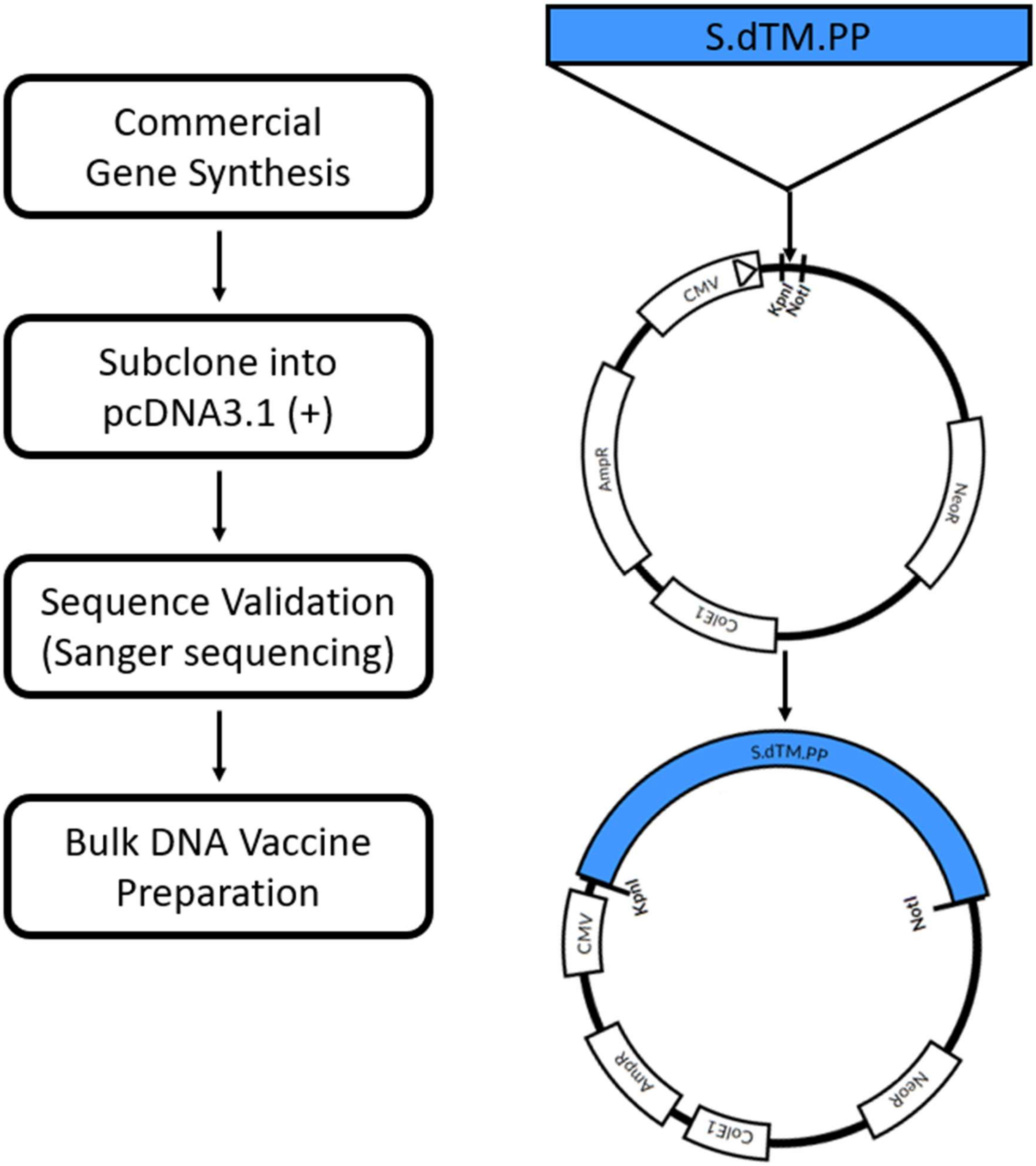

Supplemental Figure 1: DNA Vaccine Construction. Commercially synthesized DNA sequences were subcloned into pcDNA3.1 (+) using KpnI and NotI restriction sites. DNA vaccines were sequenced validated before bulk preparation.

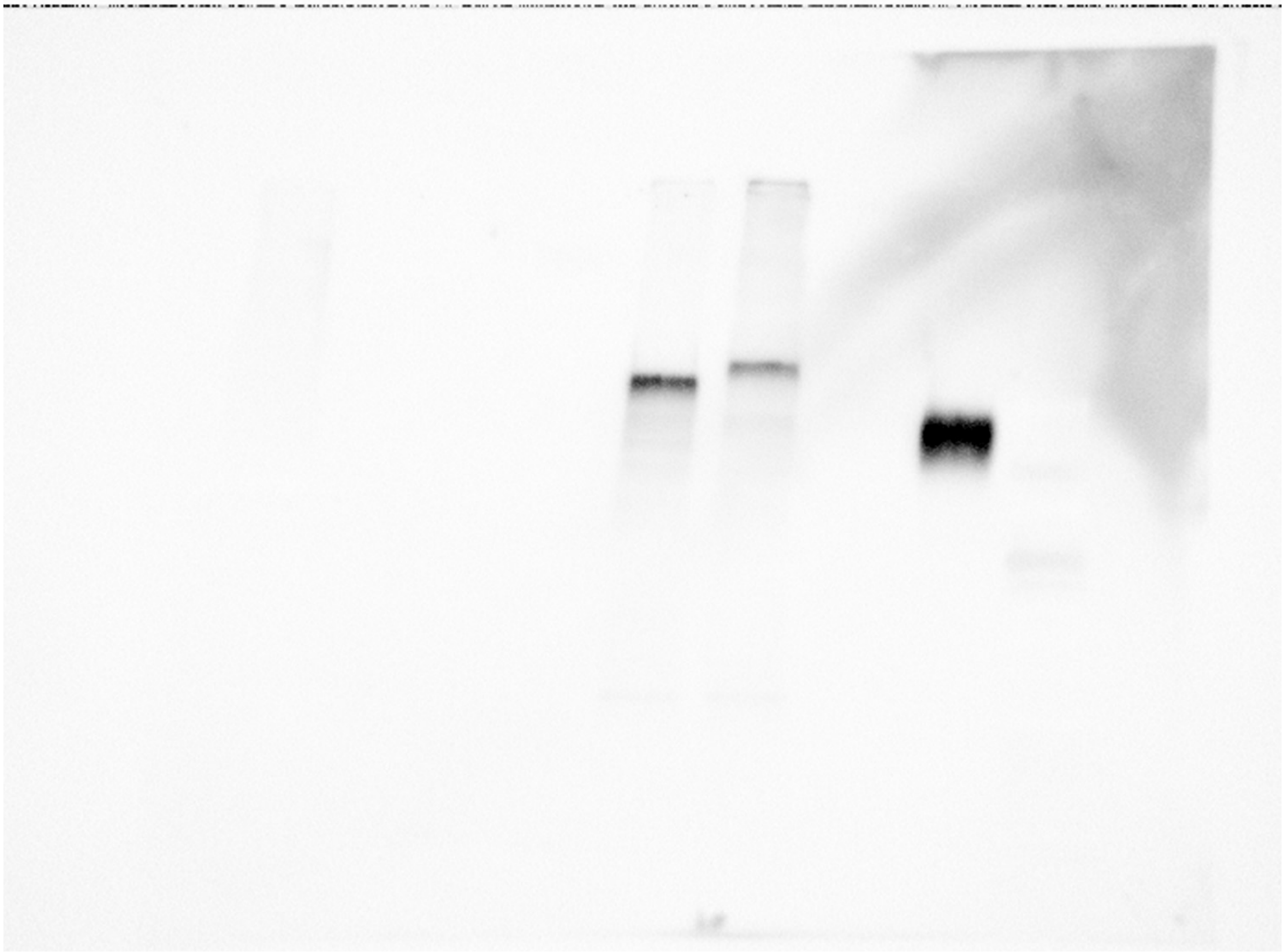

Supplemental Figure 2: Spike Western Blot Images. SARS-CoV-2 (2019-nCoV) Spike Antibody, Rabbit PAb, Antigen Affinity Purified (Cat: 40591-T62, Sino Biologics). SARS-CoV-2 (2019-nCoV) Spike S1+S2 ECD-His recombinant protein (Sino Biological) used as a positive control. Chemiluminescence image of entire membrane.

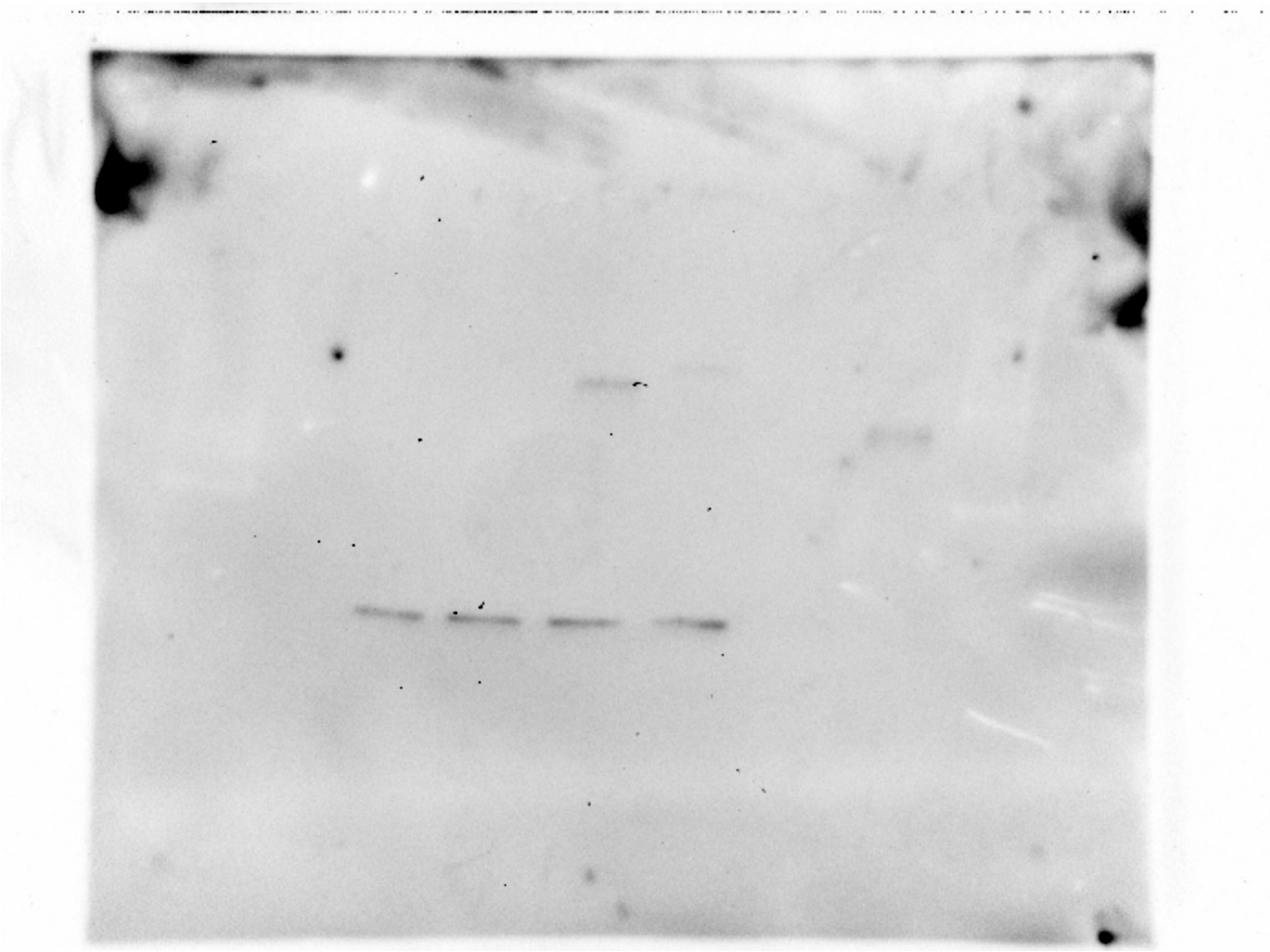

Supplemental Figure 3: B-actin Western Blot Image. β-Actin Antibody (Cat: 4967, Cell Signaling). Chemiluminescence image of entire membrane.

Supplemental Table 1. DNA Vaccines against SARS-CoV-2 in clinical trials. S – Spike. N – Nucleoprotein. ID – Intradermal. IM – Intramuscular. EP- Electroporation. https://clinicaltrials.gov/ accessed November 16 2021.

Vaccine Name	Vaccine Antigen	Administration Route (Doses)	Administration Device	Clinical Trial Phase	Clinical Trial Identifier
INO-4800	S	ID (2)	EP	II/III	NCT04642638
AG0302-COVID19	S	IM (2)	N/A	II/III	NCT04655625
GX-19N	S and N	IM (2)	EP	II/III	NCT05067946
VB10.2129 (C1) and VB10.2210	S or multi-antigen	IM (1 or 2)	N/A	I/II	NCT05069623
Covigenix VAX-001	S	IM (2)	N/A	I/II	NCT04591184
GLS-5310	S	ID (2)	N/A	I/II	NCT04673149
GX-19	S	IM (2)	EP or needle-free injection	I/II	NCT04445389
COVID-eVax	S	IM (1 or 2)	EP	I/II	NCT04788459
AG0301-COVID19	S	IM (or 2)	N/A	I/II	NCT04463472
CORVAX-12	S	IM (1)	EP	I	NCT04627675
COVIGEN	S	IM/ID (2)	needle-free injection	I	NCT04742842
COVIDITY	S and N	IM/ID (2)	needle-free injection	I	NCT05047445
